# Bacterial and fungal microflora in surgically removed lung cancer samples

**DOI:** 10.1186/1749-8090-6-137

**Published:** 2011-10-14

**Authors:** Panagiotis Apostolou, Aggeliki Tsantsaridou, Ioannis Papasotiriou, Maria Toloudi, Marina Chatziioannou, Gregory Giamouzis

**Affiliations:** 1 Research Genetic Cancer Centre Ltd (R.G.C.C. Ltd), Filotas, Florina, Greece; 2 Department of Cardiovascular and Thoracic Surgery, Larissa University Hospital, Larissa, Greece; 3 Cardiology Department, Larissa University Hospital, Larissa, Greece

**Keywords:** lung cancer, bacteria, fungi, reverse-transcription polymerase chain reaction

## Abstract

**Background:**

Clinical and experimental data suggest an association between the presence of bacterial and/or fungal infection and the development of different types of cancer, independently of chemotherapy-induced leukopenia. This has also been postulated for the development of lung cancer, however the prevalence and the exact species of the bacteria and fungi implicated, have not yet been described.

**Aim:**

To determine the presence of bacterial and fungal microflora in surgically extracted samples of patients with lung cancer.

**Materials and methods:**

In this single-center prospective, observational study, tissue samples were surgically extracted from 32 consecutive patients with lung cancer, and reverse-transcription polymerase chain reaction (RT-PCR) was used to identify the presence of bacteria and fungi strains.

**Results:**

The analysis of the electrophoresis data pointed out diversity between the samples and the strains that were identified. Mycoplasma strains were identified in all samples. Strains that appeared more often were Staphylococcus epidermidis, Streptococcus mitis and Bacillus strains, followed in descending frequency by Chlamydia, Candida, Listeria, and Haemophilus influenza. In individual patients Legionella pneumophila and Candida tropicalis were detected.

**Conclusions:**

A diversity of pathogens could be identified in surgically extracted tissue samples of patients with lung cancer, with mycoplasma strains being present in all samples. These results point to an etiologic role for chronic infection in lung carcinogenesis. Confirmation of these observations and additional studies are needed to further characterize the etiologic role of inflammation in lung carcinogenesis.

## Introduction

Lung cancer is the most common cancer worldwide, with 1.35 million incident cases annually, and consists one of the leading causes of mortality worldwide [1]. In addition to cigarette smoking, the major lung cancer risk factor [1], recent studies underscore an etiologic role for chronic pulmonary infection in lung carcinogenesis, acting either independently or as a cofactor to tobacco smoke in increasing lung cancer risk [2-5]. Experimental and clinical data correlate cancer development with the presence of certain pathogens, independently of chemotherapy-induced leucopenia [6-8]. Indeed, mycoplasma is one of the most often observed pathogen in lung carcinomas [9], and it has been postulated that mycoplasma-infected cells have a higher ability to metastasize in vivo than non-mycoplasma-infected cells [10]. Very similarly, the bacterium Chlamydia pneumoniae, a common cause of community-acquired pneumonia, has been implicated in lung carcinogenesis [11-16]. Staphylococcus strains likewise have been observed in many cases of patients with lung cancer [6,7,17-19]. Other studies have demonstrated the presence of colonies in respiratory tract in patients with cancer [19]; Haemophilus influenza [6,7,19-21] and Candida albicans [7,20-22] have been found in patients with lower respiratory tract malignancies. Legionella pneymophila has been diagnosed in patients with cancer [23], as well as strains of Bacillus [7], Listeria [24], and Streptococcus [6,7,17,19,25].

Importantly, previous retrospective and prospective studies have relied on serologic characterization of chronic bacterial and fungal infections [14]. To the best of our knowledge, the prevalence of bacterial and/or fungal infection in surgically extracted samples of patients with lung cancer has not been previously reported. The aim of the present study, therefore, was to determine the presence of bacterial and fungal microflora in surgically removed tissue samples of patients with lung cancer, by using PCR methods and special primers.

## Materials and methods

In this single-center prospective, observational study, tissue samples were surgically removed from 32 consecutive patients with lung cancer. The samples were maintained in RPMI culture medium (Sigma, R0883, Germany). The tissue was dissociated and 2 ml Trypsin - 0,25% EDTA (Invitrogen, 25200-072, California) was added in order to detach the cells. The trypsin has been inactivated by using FBS (Invitrogen, 10106-169, California) and cells were centrifuged at 1,200 rpm for 10 min. Then cells were incubated in 25 cm^2 ^flasks (Orange Scientific, 5520200, Belgium) at 37°C, in a 5% CO2 atmosphere, until well developed. RNA was extracted using TRIZOL (Invitrogen, 15596-026, California) and was used as a template to generate cDNA using the First strand cDNA synthesis kit (Fermentas, K1612, Canada). The First strand cDNA was used as a template for the Gradient-PCR reaction, which was performed using GoTaq Flexi polymerase (Promega, M8305, USA). Primers have been designed with Gene Expression 1.1 software. The PCR conditions were set as follows: initial denaturation at 95°C for 10 min to activate the polymerase, 35 cycles of denaturation at 94°C for 45 sec, followed by annealing at 52-58°C for 45 sec and an extension step at 72°C for 60 sec. A final extension step was performed at 72°C for 10 min. The PCR products were then separated on 1.5% agarose gel (Merck, 1012360500, USA) stained with GelGreen (Gentaur, 41005, Belgium), and finally observed under UV-light. A 100-bp ladder (Promega, G2101, USA) was used as marker.

This study was in compliance with the Helsinki Declaration. All participants gave informed consent and the study was approved by the institutional board review.

## Results

**Figure 1:**
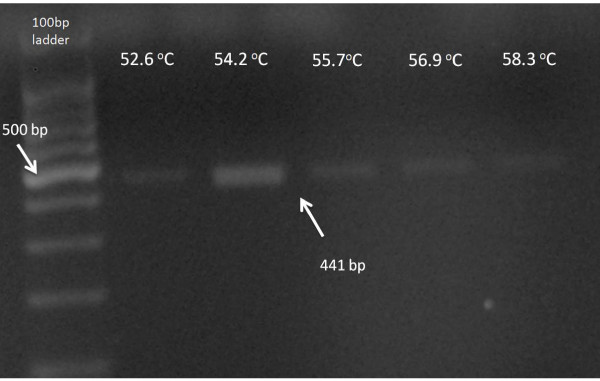
**Electrophoresis results for Mycoplasma strains**.

Table 1 shows the primer pairs that were used in PCR to identify the specific pathogen strains. Table 2 presents the frequency of different species and strains in the samples that were examined. The analysis of the electrophoresis data pointed out diversity between the samples and the strains that were identified in them. Mycoplasma strains were identified in all samples (Figure 1 demonstrates electrophoresis results for Mycoplasma strains). Strains that appeared more often were Staphylococcus epidermidis, Streptococcus mitis and Bacillus strains, followed in descending frequency by Chlamydia, Candida, Listeria, and Haemophilus influenza. In individual patients Legionella pneumophila and Candida tropicalis were detected.

**Table 1:** Primer pairs that have been used in PCR

Organism	Species	Forward Primer (5'-3')	Reverse Primer (5'-3')	PCR Product (bp)
Treponema	pallidum	AATGCGGTGGCGTAGCGATAC	TTTTGCGGTTTGCTCCACTTC	275
	
	denticola	AGGGATATGGCAGCGTAGCAATA	CGTCCTCCCTTACGGGTTAGACT	453
	
	vincentii	GCGGTATGTAAGCCTGGTGTGAA	TTTGCTTTGGCACTGAAGCTCTT	277

Neisseria	meningitidis	AAGTCGGACGGCAGCACAGA	TCAGCCGCTGATATTAGCAACAG	421

Legionella	pneumophila	AAGATTAGCCTGCGTCCGATTAG	AACCCTCCTCCCCACTGAAAGT	232

Borrelia	burgdorferi	CATGCAAGTCAAACGGGATGTA	GACCTTCTTCATTCACGCAGTG	361
	americana			
	valaisiana			
	garinii			
	recurrentis			
	hispanica			
	duttonii			
	lusitaniae			
	spielmanii			

Listeria	grayi	TCTTGACATCCTTTGACCACTCTG	TGCACCGGCAGTCACTTTAGAG	157
	innocua			
	monocytogenes			
	welshimeri			

Helicobacter	pylori	GATTGGCTCCACTTCGCAGTA	GGCGACCTGCTGGAACATT	560
	pullorum			
	equorum			
	canadensis			

Staphylococcus	aureus	AGGCGACTTTCTGGTCTGTAACTG	CCGAAGGGGAAGGCTCTATCT	307

Haemophilus	parasuis	CCTTGGGAAAATACTGACGCTCAT	TCCCGAAGGCACACTCTCAT	297

Chlamydia	muridarum	TGTTTAGTGGCGGAAGGGTTAG	CCGTCCATTGCGAAAGATTC	304
	trachomatis			

Bacillus	pumilus	TGCAAGTCGAGCGGACAGA	TCCCAGTCTTACAGGCAGGTTAC	91
	aerophilus			
	licheniformis			
	amyloliquefaciens			
	subtilis			

Bacillus II	anthracis	CGGCTTCGGCTGTCACTTATG	TCAGCACTAAAGGGCGGAAAC	655
	cereus			
	thuringiensis			

Mycoplasma	pneumoniae	GAGGCGAACGGGTGAGTAACA	CGCGACTGCTGGCACATAGT	441
	pirum			
	gallicepticum			
	genitalium			
	amphoriforme			

Leptospira	borgpetersenii	GGATAGCCCCGAGAGGTCATA	CCATCATCACATCGCTGCTTAT	299

Leptospira	meyeri	CGAATGTGACGGTTCCTGGTAG	TTCGCCCATTGAGCAAGATT	210
	biflexa			
Staphylococcus	epidermidis	GTGAAAGACGGTTTTGCTGTCAC	CGGATAACGCTTGCCACCTAC	359

Streptococcus	mitis	GGAGCTTGCTCTTCTGGATGAG	GAGCCGTTACCCCACCAACT	197

Leptospira	interrogans	CAGCCTGCACTTGAAACTATGTG	ATAGTCCCCAGGCGGTCTACT	266

Brachyspira	hyodysenteriae	TGCCGTAGAGTGGGGGATAA	CCGCAGGCTCATCGTAAAG	109
	aalborgi			
	intemedia			
	alvinipulli			
	innocens			
	suanatina			

Haemophilus	influenzae	CTTGCTTTCTTGCTGACGAGTG	TCTCAGTCCCGCACTTTCATC	129

Candida I	albicans	CCAGCCGAGCCTTTCCTTCT	TACCCCCGACCGTCCCTATT	187
	parapsilosis			
	dubliensis			

Candida	tropicalis	CGGTCGGGGGTATCAGTATTC	ATACTCGCTGGCTCCGTCAGT	622

**Table 2:** Prevalence by different species and strains

Pathogen	Strain	Prevalence (%)
Legionella	pneumophila	3.125
Listeria	grayi	9.375
	innocua	
	monocytogenes	
	welshimeri	
Chlamydia	muridarum	12.5
	trachomatis	
Bacillus	pumilus	28.125
	aerophilus	
	licheniformis	
	amyloliquefaciens	
	subtilis	
Staphylococcus	epidermidis	25
Streptococcus	mitis	21.875
Haemophilus	influenzae	6.25
Mycoplasma	pneumonia	100
	pirum	
	gallicepticum	
	genitalium	
	amphoriforme	
Candida	albicans	12.5
	parapsilosis	
	dubliensis	
Candida	tropicalis	3.125

## Discussion

Lung cancer is the most common cancer worldwide and is a leading causes of mortality worldwide [1]. Many recent studies have underscored the etiologic role of chronic pulmonary infection in lung carcinogenesis, concluding that inflammation increases the risk for incident lung cancer [2-5]. Numerous studies on lung cancer have pointed out the appearance of Mycoplasma strains in patients and suggest association of infection with tumorigenesis; it has been postulated that mycoplasma-infected cells have a higher ability to metastasize in vivo than non-mycoplasma-infected cells [10]. Candida species have been isolated from patients with lower respiratory tract infection [7,20-22]. Haemophilus influenza [6,7,19-21], Staphylococcus epidermidis [6,7,17-19], Streptococcus species [6,7,17,19,25], Legionella pneymophila [23], as well as strains of Bacillus [7], Listeria [24] and Streptococcus [6,7,17,19,25] have been also identified in patients with different pulmonary diseases. Very similarly, the bacterium Chlamydia pneumoniae, a common cause of community-acquired pneumonia, has been implicated in lung carcinogenesis [11-16]. A recent meta-analysis by Zhan et al. [16] of 12 studies involving 2595 lung cancer cases and 2585 controls from four prospective studies and eight retrospective studies, was conducted to analyze the association between C. pneumoniae infection and risk of lung cancer. Overall, people exposed to C. pneumoniae infection had an odds ratio (OR) of 1.48 (95% confidence interval (CI), 1.32-1.67) for lung cancer risk, relative to those not exposed. Of interest, a higher titre was an even better risk prognosticator (OR for IgA ≥64 cutoff group, 2.35; 95% CI, 1.88-2.93; OR for IgA ≥16 cutoff group, 1.22; 95% CI, 1.06-1.41).

These data strongly support the idea that lung cancer is a biofilm associated chronic infection. Biofilms are microorganism populations organized in a form of colonies using self-produced extracellular matrix that works as infrastructure material. The vast majority of the micro-"colonists" establish biofilms on any inert or diseased biological surface. They adhere to each other, divide, cooperate, and, progressively, their bio-mass grows, matures and finally disperses. It resembles malignant behavior (tumors composed by cancer cells and by stroma cells-monocytes, lymphocytes, microvessels, can metastasize). Therefore, many researchers imply that lung malignancies are communities of diverse pathogens resistant to antibiotics.

One of the major limitations in most of the previous studies was the use of serologic characterization to identify chronic bacterial or fungal infections [14]. This has resulted in conflicting results and great variability in relative risk estimations among seropositive individuals [14,15,26-29]. This wide variability could also reflect the retrospective nature of most of the studies, the small sample sizes, or inadequate adjustment for confounding factors [14]. New techniques, such as PCR-RFLPs, Matrix-assisted laser desorption ionization time-of-flight mass spectrometry (MALDI-TOF MS) and microcolony methods allow examination and analysis of microbial communities [30,31]. Analyzing the constituents of microbial biofilms responsible for lung disease may help us discover novel strategies to control malignancies.

To the best of our knowledge, the prevalence of bacterial and/or fungal infection in surgically extracted samples of patients with lung cancer has not been previously reported. Therefore, the main purpose of the present study was to determine the presence of bacterial and fungal microflora in surgically removed tissue samples of patients with lung cancer, by using PCR methods and special primers. In this study, specific primers were designed in order to amplify as many different strains of microorganisms. Pairs of primers that were designed were capable of amplifying Treponema, Neisseria, Legionella, Borrelia, Listeria, Helicobacter, Staphylococcys, Haemophilus, Bacillus, Leptospira, Streptococcus, Mycoplasma, Candida and Brachyspira species. It is worth noting that Mycoplasma species were observed in all samples. Staphylococcus epidermidis and Streptococcus mitis were almost seen in one quarter of patients. Neither Treponema strains nor Leptospira, Helicobacter, and Staphylococcus aureus strains were observed in this study.

## Conclusion

A diversity of pathogens could be identified in surgically extracted tissue samples of patients with lung cancer, with mycoplasma strains being present in all samples. These results point to an etiologic role for chronic infection in lung carcinogenesis. Confirmation of these observations and additional studies are needed to further characterize the etiologic role of inflammation in lung carcinogenesis, thus making it possible to apply new therapeutic modalities.

## Competing interests

The authors declare that they have no competing interests.

## Authors' contributions

PA carried out the molecular studies and drafted the manuscript. AT participated in the design of the study and collected all tissue samples. IP participated in the design of the study and coordination. MT carried out the molecular studies and drafted the manuscript. MC carried out the molecular studies and drafted the manuscript. GG performed the statistical analysis and drafted the manuscript.

All authors read and approved the final manuscript.
